# The Effects of Sidt2 on the Inflammatory Pathway in Mouse Mesangial Cells

**DOI:** 10.1155/2020/3560793

**Published:** 2020-05-28

**Authors:** Hui Sun, Jia-ming Ding, Hui-hao Zheng, Kang-jia Lv, Yun-fei Hu, Ying-hui Luo, Xue Wu, Wen-jun Pei, Li-zhuo Wang, Ming-cai Wu, Yao Zhang, Jia-lin Gao

**Affiliations:** ^1^Department of Endocrinology and Genetic Metabolism, Yijishan Hospital of Wannan Medical College, Wuhu 241002, China; ^2^Institute of Endocrine and Metabolic Diseases, Yijishan Hospital of Wannan Medical College, Wuhu 241002, China; ^3^School of Clinical Medicine, Wannan Medical College, Wuhu 241002, China; ^4^School of Medical Imaging, Wannan Medical College, Wuhu 241002, China; ^5^School of Pharmacy, Wannan Medical College, Wuhu 241002, China; ^6^Anhui Province Key Laboratory of Biological Macro-molecules Research, Wannan Medical College, Wuhu 241001, China; ^7^Department of Biochemistry and Molecular Biology, Wannan Medical College, Wuhu 241002, China

## Abstract

In patients with chronic kidney disease, the abnormal activation of inflammatory pathways is usually an important factor leading to renal fibrosis and further deterioration of renal function. Finding effective intervention targets of the inflammatory signaling pathway is an important way to treat chronic kidney disease. As a newly discovered lysosomal membrane protein, the correlation between SID1 transmembrane family member 2 (Sidt2) and the inflammatory signaling pathway has not been reported. The aim of this study was to investigate the effect of Sidt2 on inflammation by inhibiting the expression of the *Sidt2* gene in a mouse mesangial cell line mediated by a lentiviral CRISPR/Cas9 vector. Hematoxylin and eosin staining and microscopy found that the mesangial cells lost their normal morphology after inhibiting the expression of Sidt2, showing that the cell body became smaller, the edge between the cells was unclear, and part of the nucleus was pyknotic and fragmented, appearing blue-black. The expressions of IKK *β*, p-IKK *α*/*β*, NF-*κ*B p65, p-NF-*κ*B p65, p-I*κ*B*α*, I*κ*B*α*, and TNF-*α* in the NF-*κ*B pathway of the *Sidt2*^−/−^ group were higher than those of the *Sidt2*^+/+^ group. p-Jak2 and IL6 increased in the Jak/Stat pathway, and p-ERK and p-P38 increased in the MAPK pathway. The expressions of IKK *β*, p-IKK *α*/*β*, NF-*κ*B p65, p-NF-*κ*B p65, p-I*κ*B*α*, I*κ*B*α*, and TNF-*α* in the NF-*κ*B pathway of the *Sidt2*^+/+^+LPS group were significantly higher than those in the *Sidt2*^+/+^ group. The expressions of IKK *β*, p-IKK *α*/*β*, NF-*κ*B p65, p-NF-*κ*B p65, p-I*κ*B*α*, I*κ*B*α*, and TNF-*α* in the *Sidt2*^−/−^+LPS group were higher than those in the *Sidt2*^−/−^ group. The expressions of p-IKK *α*/*β*, NF-*κ*B p65, p-NF-*κ*B p65, p-I*κ*B*α*, I*κ*B*α*, and TNF-*α* in the *Sidt2*^−/−^+LPS group were higher than those in the *Sidt2*^+/+^+LPS group. In the Jak/Stat pathway, the protein expressions of p-Jak2 and IL6 in the *Sidt2*^+/+^+LPS group were higher than those in the *Sidt2*^+/+^ group. The expressions of p-Jak2 and IL6 in the *Sidt2*^−/−^+LPS group were higher than those in the *Sidt2*^−/−^ group. The expressions of p-Jak2 and IL6 in the *Sidt2*^−/−^+LPS group were higher than those in the *Sidt2*^+/+^+LPS group. The expressions of p-JNK, p-ERK, p-P38, and ERK in the MAPK pathway in the *Sidt2*^+/+^+LPS group were higher than those in the *Sidt2*^+/+^ group. The expressions of p-JNK, p-ERK, p-P38, and ERK in the *Sidt2*^−/−^+LPS group were higher than those in the *Sidt2*^−/−^ group. The expressions of p-JNK, p-ERK, p-P38, and ERK in the *Sidt2*^−/−^+LPS group were higher than those in the *Sidt2*^+/+^+LPS group. These data suggested that deletion of the *Sidt2* gene changed the three inflammatory signal pathways, eventually leading to the damage of glomerular mesangial cells in mice.

## 1. Introduction

Inflammation plays a key role in many chronic diseases, including cardiovascular disease, cancer, chronic kidney disease, and diabetes [1]. In the inflammatory regulatory network of the body, inflammation plays an important role, mainly by affecting genes that play a key regulatory role in signal transduction pathways. The activation of inflammatory signal pathways changes the expression and activity of inflammation-related downstream effector proteins, and these participate in the inflammatory process of chronic diseases. In recent years, the incidence of chronic kidney disease (CKD) is increasing, but its pathogenesis is complex. Renal interstitial fibrosis (RIF), renal hemodynamic changes, inflammatory signaling pathway activation, and genetic factors are involved in the occurrence and development of CKD, and inflammatory signaling pathways are considered to be the most important [2]. With the development of the study of CKD, three inflammatory signal pathways, NF-*κ*B, Jak/Stat, and MAPK, have been identified as most closely related to the occurrence and development of CKD [3, 4]. Abnormal activation of inflammatory signaling pathways can accelerate RIF, which is not only a common pathophysiological manifestation of the occurrence and development of CKD but also plays a key role in the development of end-stage kidney disease [5, 6]. Therefore, an in-depth study of inflammatory signal transduction pathways and the key signal transduction molecules in CKD is of great significance for intervention of the progression of CKD from the perspective of inflammation.

The hypothesis of a “suicide sac,” as put forward by deDuve, the discoverer of the lysosome, has been accepted by most scholars. During inflammation, a series of morphological changes causing degeneration and necrosis of tissue cells is closely related to the lysis of lysosomes in the cytoplasm and the release of lysosomal substances. Lysosomes contain a variety of proteolytic enzymes, including lipases and phosphatase, which not only autolyze the injured cells themselves but also cause degeneration and necrosis of the surrounding tissues and cells [7, 8]. Lysosomes can regulate the immune response by regulating the secretion of inflammatory cytokines (such as IL-1 *β*, IL-18, and TNF-*α*). Through positive and negative regulatory pathways, lysosomes maintain the balance of the inflammatory response in cells and organelles. Abnormal overexpression of lysosomal and cytoskeletal pathway components leads to changes in inflammatory signaling pathway activity [9]. In the past, it was generally believed that lysosomal membrane proteins were only the means of maintaining membrane integrity and the lysosomal environment. However, further studies have shown that lysosomal membrane proteins are not only involved in the transport of a variety of substances but are also involved in multiple signal pathways. So far, more than 50 kinds of lysosomal membrane proteins have been found, including LAMP1, LAMP2, sialin, NPC1, mucolipin, TMEM9B, and SID1 transmembrane family member 2 (Sidt2). Previous studies have shown that lysosomal membrane proteins (such as TMEM9B) are key components of inflammatory signaling pathways and participate in the activation of NF-*κ*B and MAPK pathways [10]. As a newly identified lysosomal membrane protein, the effect of Sidt2 on inflammatory signaling pathways is unknown. Sidt2 is highly expressed in the kidney, and we have previously found that the kidneys of *Sidt2* knockout mice showed damage of the glomerular filtration membrane barrier (data published separately). Based on these data, in this study, we determined the effects of Sidt2 on the inflammatory signal pathway of kidney-derived cells and identified potential novel targets for blocking the renal inflammatory signal pathway. These findings will be further helpful for our understanding of the lysosomal membrane protein functions.

## 2. Materials and Methods

### 2.1. Cell Culture and Treatment

The mouse glomerular mesangial SV40 MES 13 cells were obtained from the Cell Culture Bank of the Chinese Academy of Sciences. The cells were propagated in a medium recommended by the cell bank, which contained DMEM (Gibco, Cat. No. 12800017, added NaHCO_3_ 1.5 g/L), 71.25%, F-12 medium (Gibco, Cat. No. 21700075, added L-glutamine 150 mg/L, NaHCO_3_ 1.5 g/L), 23.75%, 14 mM HEPES, fetal calf serum (Gibco, USA), 10%. Maintain standard culture conditions (gas phase: air, 95%; carbon dioxide, 5%; temperature: 37°C). SV40 MES 13 cells were cultured in 6-well culture dishes until they reached approximately 50% confluence and were then stimulated with different concentrations of LPS (Beyotime, China) for 12 hours.

### 2.2. Knockout of *Sidt2* Gene by CRISPR/Cas9 Gene Editing Lentiviral Vector System

First, the SV40 MES 13 cells were resuscitated. The cells were subcultured every 3 or 4 days at the appropriate cell density. The CRISPR/Cas9 lentivirus system was used to generate mouse *Sidt2* KO SV40 MES 13 cell lines. The cells were selected with medium containing puromycin after transfection to eliminate nontransfected cells. The details referenced [11]. The reverse transcription-polymerase chain reaction (RT-PCR) and western blot assays were used to detect the mRNA and protein expression of Sidt2.

### 2.3. Reverse Transcription-Polymerase Chain Reaction (RT-PCR) Analysis

Total RNA from SV40 MES 13 cells were isolated using Trizol reagents (Invitrogen, USA). Reverse transcription was performed using high-capacity complementary DNA (cDNA) reverse transcription kit following manufacturer's instructions (Thermo Fisher Scientific, China). *β-Actin* was used as loading control. The *Sidt2* primer was used as 5′-ATGTGGTGGGTGTAGTGTAGTGAAAG-3′ and 5′-AGATACACCACCACCACCATCAC-3′; *β-actin* was used as 5′-GACAGGATGCAGAAGAGATTACT-3′ and 5′-TGATCCACATCTGCTGGAAGGT-3′.

### 2.4. HE Staining

The adherent cells were digested with trypsin (Biosharp BL526A, China), incubated on cell climbing slices for 24 hours, and rinsed three times in PBS at 4°C. The cells were then fixed with prechilled 4% paraformaldehyde for 30 min at 4°C. Hematoxylin and eosin staining was performed and observed by inverted phase-contrast microscopy.

### 2.5. Western Blotting Analysis of the Protein Expression

Cell samples were lysed in a RIPA lysis buffer (Beyotime, China) containing PMSF (Beyotime, China) at 4°C for 20 minutes, sonicated two times for 1 minute, and centrifuged at 12000 × g for 15 minutes at 4°C, and the supernatant was collected. The protein concentration was determined by Thermo Scientific NanoDrop2000c. Total protein (80 mg) was run on (8%-15%) gel SDS-PAGE and transferred to a polyvinylidene fluoride membrane (Milibo, USA). The membrane was incubated overnight in the desired primary antibody at 4°C, then the secondary antibody (Beyotime, China) was incubated at room temperature for 1.5 hours, and finally, ECL chemiluminescence was used for imaging. Density analysis of all western blots was performed using ImageJ v1.46 software (http://imagej.nih.). Antibodies used included *β*-actin (Sigma, USA), Sidt2 (Sigma, USA), TNF-*α* (Proteintech, USA), IL6 (Proteintech, USA), NF-*κ*B p65 (CST, USA), p-NF-*κ*B p65 (CST, USA), p-ERK (CST, USA), p-SAPK/JNK (Thr183/Tyr185) (CST, USA), p-Ikk*α*/*β* (S176/180) (CST, USA), I*κ*B*α* (CST, USA), p-I*κ*B*α* (S32) (CST, USA), p-P38MAPK (CST, USA), p-STAT3 (CST, USA), Ikk*β* (ABclonal, USA), p-JAK2 (Thy1007/1008) (ABclonal, USA), and ERK (ABclonal, USA).

### 2.6. Statistical Analysis

The results of data are presented as the means ± SEM of at least three independent experiments. Unpaired *t*-test was used to assess statistical comparison between two groups. GraphPad Prism 8.0 software was used for statistical analyses. Values of *p* < 0.05 were considered significant differences.

## 3. Results

### 3.1. Establishment of a *Sidt2* Knockout Cell Model and Morphological Observations

**Figure 1:**
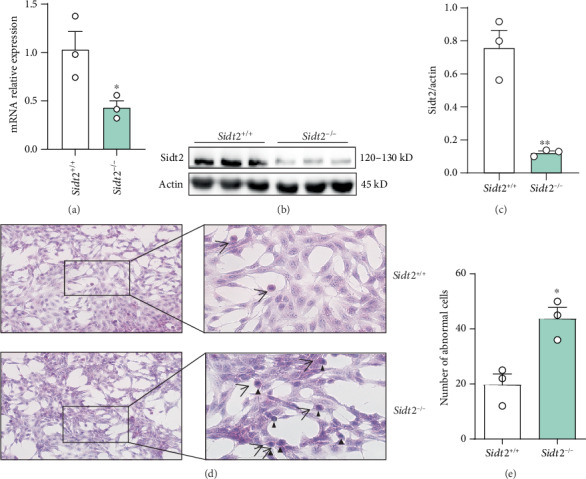
Establishment of a *Sidt2* knockout cell model and morphological observation. (a) The level of mRNA in the *Sidt2*^−/−^ group was significantly lower than that in the *Sidt2*^+/+^ group. (b c) The expression of Sidt2 in the *Sidt2*^−/−^ group was significantly decreased by western blotting compared with that in the *Sidt2*^+/+^ group. (d) Hematoxylin and eosin staining revealed different cellular states between the *Sidt2*^−/−^ group and the *Sidt2*^+/+^ group. The cell body of the *Sidt2*^−/−^ group was significantly smaller than that of the *Sidt2*^+/+^ group (arrow), and the nuclear pyknosis and fragmentation were blue-black (triangle). *n* ≥ 3, compared with the *Sidt2*^+/+^ group, ∗*p* < 0.05 and ∗∗*p* < 0.01.

The *Sidt2* knockout model of SV40 MES 13 cells in culture was confirmed by qPCR and western blotting at the mRNA and protein levels, respectively. The results of qPCR showed that the level of mRNA in the *Sidt2*^−/−^ group was significantly lower than that in the *Sidt2*^+/+^ group (Figure 1(a)). This was further verified by western blotting results showing that the level of Sidt2 protein was significantly lower than that in the *Sidt2*^+/+^ group (Figures 1(b) and 1(c)), confirming that the *Sidt2* knockout cell model was successfully constructed. Hematoxylin and eosin (HE) staining (Figure 1(d); ×20) was performed on the *Sidt2*^−/−^ group and the *Sidt2*^+/+^ group samples. The results showed that the cell bodies of the *Sidt2*^−/−^ group cells were significantly smaller than those of the *Sidt2*^+/+^ group cells (Figure 1(d), arrow). The edges between cells were unclear, the cells lost their normal morphology, parts of the nuclei were pyknotic and fragmented (see the blue-black nuclei shown in Figure 1(d), triangle), and the proportion of abnormal cells was significantly higher than that of the control group (Figure 1(e)).

### 3.2. Inhibition of *Sidt2* Gene Expression in Mouse Glomerular Mesangial Cells Increased the Level of Inflammation

**Figure 2:**
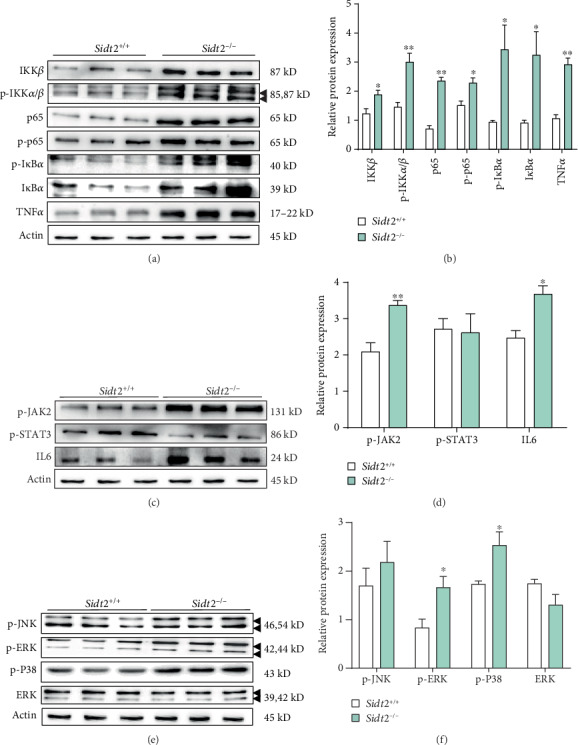
Inhibition of *Sidt2* gene expression in mouse glomerular mesangial cells increased the level of inflammation. (a, b) Compared with the *Sidt2*^+/+^ group, the protein expression of the NF-*κ*B pathway including IKK *β*, p-IKK *α*/*β*, NF-*κ*B p65, p-NF-*κ*B p65, p-I*κ*B*α*, I*κ*B*α*, and TNF-*α* in the *Sidt2*^−/−^ group increased significantly. (c, d) The protein expression of the JAK/STAT pathway including p-Jak2 and IL6 in the *Sidt2*^−/−^ group was significantly higher compared with that in the *Sidt2*^+/+^ group. (e, f) The protein expression of the MAPK pathway including p-ERK and p-p38 in the *Sidt2*^−/−^ group was significantly higher than that in the *Sidt2*^+/+^ group. *n* ≥ 3, ∗*p* < 0.05, ∗∗*p* < 0.01.

We extracted total cellular proteins and used western blot analysis to detect the levels of NF-*κ*B, Jak/Stat, and MAPK pathway proteins. The results showed that the protein levels of IKK *β*, p-IKK *α*/*β*, NF-*κ*B p65, p-NF-*κ*B p65, p-I*κ*B*α*, I*κ*B*α*, and TNF-*α* in the *Sidt2*^−/−^ group were significantly higher than those in the *Sidt2*^+/+^ group (Figures 2(a) and 2(b)). Regarding the JAK/STAT pathway, the expressions of p-Jak2 and IL6 proteins in the *Sidt2*^−/−^ group were higher than those in the *Sidt2*^+/+^ group, but there was no significant difference in p-STAT3 protein expression levels (Figures 2(c) and 2(d)). For MAPK pathway proteins, the expression levels of p-ERK and p-P38 in the *Sidt2*^−/−^ group were significantly higher than those in the *Sidt2*^+/+^ group, but there were no significant differences in p-JNK and ERK expressions (Figures 2(e) and 2(f)).

### 3.3. Changes of Inflammatory Cytokine NF-*κ*B p65 Levels in SV40 MES 13 Cells Were Stimulated by Different Concentrations of Lipopolysaccharide (LPS)

**Figure 3:**
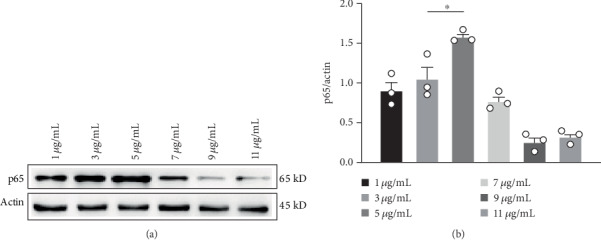
SV40 MES 13 cells were treated with different concentrations of LPS; after treatment, western blot analysis was performed to measure the protein expression levels of NF-*κ*B p65. *n* ≥ 3, ∗*p* < 0.05.

Based on the above results, we incubated LPS with SV40 MES 13 cells to establish a glomerulonephritis cell model. SV40 MES 13 cells in quiescent phase were stimulated with 1, 3, 5, 7, 9, and 11 *μ*g/mL LPS for 12 h. We extracted total cellular proteins and used western blot analysis to detect changes in NF-*κ*B p65 protein. The highest expression of NF-*κ*B p65 protein was found by western blotting assays when the concentration of LPS was 5 *μ*g/mL (Figures 3(a) and 3(b); ∗*p* < 0.05, compared with the 3 *μ*g/mL LPS group).

### 3.4. Inhibition of *Sidt2* Gene Expression Led to More Activation of Inflammatory Pathway Components in SV40 MES 13 Cells Stimulated by LPS

**Figure 4:**
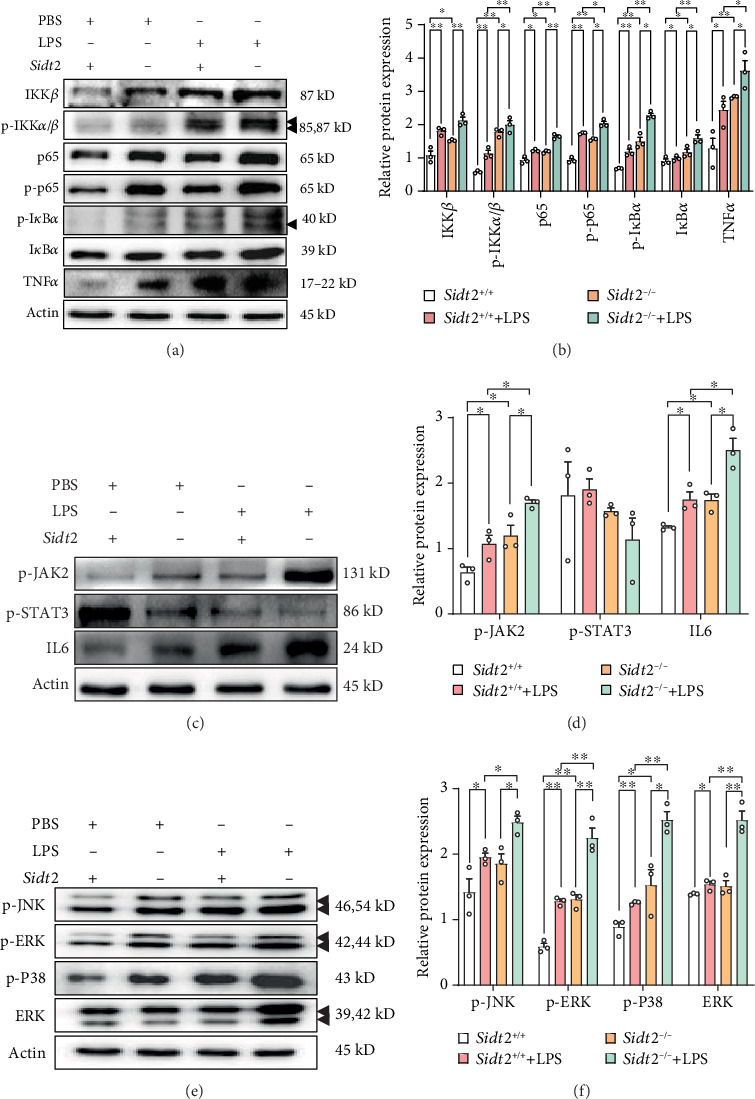
Under the stimulation of LPS, the deletion of *Sidt2* gene leads to the activation of inflammatory pathway in SV40 MES 13 cells. (a, b) After being treated with LPS for 12 hours, the protein expressions of the NF-*κ*B pathway including IKK *β*, p-IKK *α*/*β*, NF-*κ*B p65, p-NF-*κ*B p65, p-I*κ*B*α*, I*κ*B*α*, and TNF-*α* in the *Sidt2*^+/+^ group, the *Sidt2*^+/+^+LPS group, the *Sidt2*^−/−^ group, and the *Sidt2*^−/−^+LPS group were detected by western blotting. (c, d) After being treated with LPS for 12 hours, the protein expressions of the JAK/STAT pathway including p-Jak2, p-STAT3, and IL6 in the *Sidt2*^+/+^ group, the *Sidt2*^+/+^+LPS group, the *Sidt2*^−/−^ group, and the *Sidt2*^−/−^+LPS group were detected by western blotting. (e, f) After being treated with LPS for 12 hours, the protein expressions of the MAPK pathway including p-JNK, p-ERK, p-P38, and ERK in the *Sidt2*^+/+^ group, the *Sidt2*^+/+^+LPS group, the *Sidt2*^−/−^ group, and the *Sidt2*^−/−^+LPS group were detected by western blotting. *n* ≥ 3, ∗*p* < 0.05, ∗∗*p* < 0.01.

Total protein was extracted and analyzed by western blotting. Regarding the NF-*κ*B pathway proteins, the expressions of IKK *β*, p-IKK *α*/*β*, NF-*κ*B p65, p-I*κ*B*α*, I*κ*B*α*, TNF-*α*, and p-NF-*κ*B p65 in the *Sidt2*^+/+^+LPS group were significantly higher than those in the *Sidt2*^+/+^ group. The expressions of IKK *β*, p-IKK *α*/*β*, NF-*κ*B p65, p-NF-*κ*B p65, p-I*κ*B*α*, I*κ*B*α*, and TNF-*α* in the *Sidt2*^−/−^+LPS group were higher than those in the *Sidt2*^−/−^ group. The expressions of p-IKK *α*/*β*, NF-*κ*B p65, p-NF-*κ*B p65, p-I*κ*B*α*, I*κ*B*α*, and TNF-*α* in the *Sidt2*^−/−^+LPS group were higher than those in the *Sidt2*^+/+^+LPS group (Figures 4(a) and 4(b)). For the Jak/Stat pathway proteins, the protein levels of p-Jak2 and IL6 in the *Sidt2*^+/+^+LPS group were significantly higher than those in the *Sidt2*^+/+^ group. The expressions of p-Jak2 and IL6 in the *Sidt2*^−/−^+LPS group were higher than those in the *Sidt2*^−/−^ group, but the expression of p-STAT3 protein showed no significant change. The expressions of p-Jak2 and IL6 proteins in the *Sidt2*^−/−^+LPS group were higher than those in the *Sidt2*^+/+^+LPS group (Figures 4(c) and 4(d)), and the expressions of p-JNK, p-ERK, p-P38, and ERK in the MAPK pathway of the *Sidt2*^+/+^+LPS group were significantly higher than those in the *Sidt2*^+/+^ group. The protein expression levels of p-JNK, p-ERK, p-P38, and ERK in the *Sidt2*^−/−^+LPS group were higher than those in the *Sidt2*^−/−^ group. Compared with the *Sidt2*^+/+^+LPS group protein levels, the expressions of p-JNK, p-ERK, p-P38, and ERK in the *Sidt2*^−/−^+LPS group were higher than those in the *Sidt2*^+/+^+LPS group (Figures 4(e) and 4(f); ∗*p* < 0.05, ∗∗*p* < 0.01).

## 4. Discussion

NF-*κ*B is a nuclear transcription factor, which can specifically bind to the enhancer *κ*B sequence of the B cell immunoglobulin *κ* light chain gene. The NF-*κ*B pathway, especially the canonical one, dynamically regulates the response to external stimulation. NF-*κ*B signaling contains a negative feedback loop in the core network module. After extracellular stimulation, NF-*κ*B is released from I*κ*B in the cytoplasm and translocates into the nucleus, which induces the expression of target genes including I*κ*B. IKK then phosphorylates I*κ*B and induces its degradation. Activated NF-*κ*B shuttles between the nucleus and the cytoplasm (N-C oscillations) and causes the oscillation of transcription activation and inactivation [12] [13]. NF-*κ*B regulates the expression of a wide range of genes, including immune-related receptors, cytokines (such as IL-1 and MCP1), adhesion molecules (ICAM, VCAM, and E-selectin), and acute phase proteins (such as SAA). NF-*κ*B plays an important role in regulating the activation of immune cells, the development of T and B lymphocytes, apoptosis, tumorigenesis, virus replication, inflammation, and a variety of autoimmune diseases [14, 15]. Janus kinase/signal transducer and transcriptional activator (the Janus kinase/signal transducer and activator of transcriptions, i.e., JAK/STAT) signal pathway mediates a variety of biological responses, including cell proliferation, differentiation, migration, apoptosis, and immunomodulation. It is a common pathway for a variety of cytokines and growth factors used to transmit signals in cells. Therefore, it is essential in the process of growth, development, and homeostasis, including hematopoiesis, immune cell development, stem cell maintenance, and organism growth. The activation of the JAK/STAT pathway promotes the occurrence and development of a variety of diseases, including solid tumors, lymphoma, leukemia, and inflammatory diseases [16, 17]. Mitogen-activated protein kinase (MAPK) is a serine/threonine protein kinase, which is involved in cell growth, development, division, and death, as well as the recognition, transmission, and amplification of a variety of biochemical signals between cells. Different extracellular stimuli activate different MAPK signaling pathways, act on different substrates, and cause specific cellular physiological responses [18–20]. Experiments have shown that activation of inflammatory signaling pathways induces the production of various apoptosis regulatory factors, the activation of tissue inflammatory cells, and the deposition of extracellular matrices, which eventually lead to tissue cell apoptosis and necrosis, aggravation of tissue injury, and tissue fibrosis. This plays an important role in the occurrence and development of a variety of renal diseases, such as acute renal injury, renal fibrosis, diabetic nephropathy, and lupus nephritis. Reports have shown that by inhibiting inflammatory signaling pathways, renal fibrosis can be reduced, renal functions can be protected, and disease progression can be delayed [21].

Lysosome is an acidic region filled with more than 60 different types of hydrolases. As an organelle in eukaryotic cells, lysosomes are mainly involved in the degradation of macromolecules such as proteins, polysaccharides, and lipids and receive intracellular and extracellular delivery mainly through endocytosis and autophagy [22]. As early as 1979, it was reported that lysosomal protease could regulate the level of intracellular receptors by controlling the inactivation of glucocorticoid receptor-Hsp90 complex and preventing the interaction between glucocorticoid receptor and glucocorticoid [23]. In 2011, it was also reported that lysosomes maintained the stability of cytoplasmic glucocorticoid receptors and that lysosomal inhibitors cooperated with glucocorticoids to mediate the transcription of proinflammatory signals. That is, there is a negative correlation between lysosome activity and function and glucocorticoid anti-inflammatory signal transduction [24]. In addition to glucocorticoid receptor regulation, secretory lysosomes can also secrete or degrade inflammatory cytokines and their receptors to regulate immune response. The release of cytokines (such as IL-1 *β*, IL18, and TNF-*α*) can be induced by external stimuli (such as LPS and ATP) [25, 26]. In addition, lysosomal membrane proteins (such as TMEM9B) are involved in the activation of the NF-*κ*B pathway and inhibit the inflammatory response of endothelial cells, which indicate that lysosomal compartments may play an important role in inflammatory signaling network [27, 28].

In the present study, we found that at the cellular level, the morphology of glomerular mesangial cells showed significant injury after the deletion of Sidt2 protein, including nuclear pyknosis and apoptosis. Based on these morphological results, we identified three inflammatory signaling pathways, the NF-*κ*B, Jak/Stat, and MAPK pathways, at the molecular level that detected the level of cellular inflammation. By detecting the expressions of these key proteins, we found that the three inflammatory signaling pathways were activated to varying degrees after Sidt2 deletion. As the main component of the cell wall of Gram-negative bacteria, LPS regulates the secretion of various inflammatory cytokines by inducing and activating the transcription factor, NF-*κ*B [29]. Therefore, we used LPS to generate a glomerulonephritis cell model to study how LPS affects the expression of inflammatory factors and to determine the function of lysosomal membrane protein Sidt2 in inflammatory regulation. It was found that in the absence of lysosomal membrane protein Sidt2, LPS stimulation induced a further release of inflammatory cytokines, suggesting further that the elimination of Sidt2 protein leads to high sensitivity of glomerular mesangial cells to LPS stimulation and activation of the NF-*κ*B, Jak/Stat, and MAPK pathways. The upregulation of TNF-*α*, IL6, and ERK then occurs in response to LPS. Changes of these inflammatory factors further suggested that lysosomes play a vital role in inflammatory signal transduction. Similarly, lysosomal membrane proteins may also play key roles in the inflammatory signal network. In the past, however, the lysosome membrane proteins were only considered as proteins maintaining morphological integrity and lysosomal function.

## 5. Conclusion

This study was the first to characterize the important relationship between lysosomal membrane protein Sidt2 and inflammation. We found that the decreased expression of Sidt2 protein induced the activation of three inflammatory signaling pathways in glomerular mesangial cells. As intrinsic renal cells, glomerular mesangial cells have a variety of biological functions, such as maintaining the integrity of the glomerular capillary network structure and regulating the glomerular filtration rate. The activation of inflammatory pathways in glomerular mesangial cells may lead to glomerular damage and renal interstitial fibrosis. It is important to understand the function of lysosomal membrane protein Sidt2; however, the relationships between Sidt2 protein, tissue inflammation, and kidney disease remain to be further explored. At present, it is not clear whether the effects of Sidt2 on renal tissue and the inflammatory level of glomerular mesangial cells in culture are direct or indirect. In the future, we plan to establish cell and animal models of Sidt2 overexpression and further explore the specific role of Sidt2 in renal tissues and cells regarding the inflammatory pathway, to further characterize the function of Sidt2 and find novel methods to cure glomerulonephritis and prevent kidney disease.

## Data Availability

The data used to support the findings of this study are available from the corresponding author upon request.
